# The glycobiology of uropathogenic *E. coli* infection: the sweet and bitter role of sugars in urinary tract immunity

**DOI:** 10.1111/imm.13330

**Published:** 2021-05-04

**Authors:** Federico Lupo, Molly A. Ingersoll, Miguel A. Pineda

**Affiliations:** ^1^ Institute of Infection, Immunity and Inflammation University of Glasgow Glasgow UK; ^2^ Department of Immunology Institut Pasteur Paris France

**Keywords:** glycobiology, Urinary tract infections, uropathogenic *E. coli*

## Abstract

Urinary tract infections (UTI) are among the most prevalent infectious diseases and the most common cause of nosocomial infections, worldwide. Uropathogenic *E*.* coli* (UPEC) are responsible for approximately 80% of all UTI, which most commonly affect the bladder. UPEC colonize the urinary tract by ascension of the urethra, followed by cell invasion, and proliferation inside and outside urothelial cells, thereby causing symptomatic infections and quiescent intracellular reservoirs that may lead to recurrence. Sugars, or glycans, are key molecules for host–pathogen interactions, and UTI are no exception. Surface glycans regulate many of the events associated with UPEC adhesion and infection, as well as induction of the host immune response. While the bacterial protein FimH binds mannose‐containing host glycoproteins to initiate infection and UPEC‐secreted polysaccharides block immune mechanisms to favour intracellular replication, host glycans on the urothelial surface and on secreted glycoproteins prevent or limit infection by inhibiting UPEC adhesion. Given the importance of glycans during UTI, here we review the glycobiology of UPEC infection to highlight fundamental sugar‐mediated processes of immunological interest for their potential clinical applications. Interdisciplinary approaches incorporating glycomics and infection biology may help to develop novel non‐antibiotic‐based therapeutic strategies for bacterial infections as the spread of antimicrobial‐resistant uropathogens is currently threatening modern healthcare systems.

## Graphical abstract

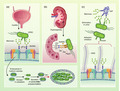

**Abbreviations**
GAG: glycosaminoglycans
GalNAc: N‐acetylgalactosamine
GlcNAc: N‐acetylglucosamine
IBCs: intracellular bacterial communities
Neu5Ac: N‐acetylneuraminic acid
SPATE: serine protease autotransporters of *Enterobacteriaceae*
THP: Tamm–Horsfall protein
UP: uroplakin plaque
UPEC: uropathogenic *E*.* coli*
UTI: Urinary tract infections

## AN INTRODUCTION TO URINARY TRACT INFECTION AND BLADDER BARRIER DEFENCES

Urinary tract infections (UTI) are among the leading causes of bacterial infections, worldwide, affecting nearly 150 million people [1]. Whereas UTI incidence steadily increases with age in men and decreases in postmenopausal women, the highest infection frequency peaks among women aged between 15 and 29 years [2]. In fact, UTI are about 40 times more prevalent in women than in men among adults under the age of 60, and the proportion of individuals with UTI, annually, is four to five times higher among women than among men in the United States [2]. This difference in incidence is less apparent in infants, children and the elderly, due in part to sex‐dependent factors, such as hormones, which impact host defences [3, 4]. The main risk factors for community‐acquired UTI are biological sex, age and history of UTI, whereas catheterization is the primary risk factor for healthcare‐associated UTI [5, 6].

The most prevalent pathogens of the urinary tract are Gram‐negative or Gram‐positive bacteria, collectively termed uropathogens [5, 6]. Accounting for about 80% of reported UTI, uropathogenic *E*.* coli* (UPEC) are the principal infectious agents of the urinary tract [5, 6]. Nonetheless, some fungi, such as *Candida* species, can proliferate in the genitourinary tract of patients with underlying urogenital abnormalities or indwelling catheters [5]. In addition, normally latent viruses, such as adenoviruses or human polyomavirus, may also colonize the lower urinary tract, potentially causing haemorrhagic cystitis in at‐risk patients, such as immunocompromised children who have undergone allogeneic bone marrow transplantation [7].

Uropathogens can colonize the gastrointestinal tract as commensal bacteria, which may be a relevant reservoir for infection [5]. UPEC may spread among people through individual behaviours and close human relationships, such as cohabitation or sexual intercourse [8, 9, 10, 11]. From the periurethral area, uropathogens ascend the urethra to the bladder to establish infection [5]. Ultimately, bacterial ascension via the ureters leads to kidney colonization, or pyelonephritis, which increases the risk of bloodstream infection [5, 12]. In men, prostate infection frequently accompanies cystitis, supporting the case for bacterial prostatitis to be classified as a UTI [13, 14].

Urinary tract infections most frequently affect the bladder, despite the virtual impenetrability of the urothelium barrier system [15]. The luminal surface of the bladder is covered with uroplakin (UP) plaques, rendering it impermeable to non‐gaseous molecules and resistant to the mechanical stresses associated with expansion and contraction of the organ [16]. UPs are integral membrane glycoproteins that assemble first as heterodimers of UP1a/UPII and UP1b/UPIIIa and then come together to form the inner and outer domains of rosette‐shaped plaques [17]. UPs are integrated into the apical membrane leaflet of hexagonally shaped umbrella or facet cells, which constitute the luminal‐facing layer of the urothelium [18]. Umbrella cells allow the bladder to accommodate variable urine volumes and maintain barrier integrity by reorganizing apical junctional rings, cytoskeleton and surface area, via Rab GTPase‐dependent exocytosis or endocytosis of a subapical reservoir of discoidal‐ and/or fusiform‐shaped vesicles [19, 20, 21, 22, 23]. In addition to UPs, proteoglycans and membrane‐tethered or secreted glycosaminoglycans (GAG) form a mucus layer that shields the urothelium from pathogens or harmful chemicals in urine [24]. The urine itself is integral to the bladder barrier because it protects the urothelium by flushing away metabolic waste and microbes. Urine also contains high concentrations of antimicrobial peptides, such as cathelicidin or lactoferrin, and opsonizing glycoproteins, such as antibodies or Tamm–Horsfall protein (THP or uromodulin), which can destroy uropathogens and block adhesion, respectively [25, 26, 27].

Glycoproteins, proteoglycans and GAG are present on outer membranes of cells as part of the cellular interface with the extracellular space [28]. The polysaccharide groups on these macromolecules, called glycans, are the master regulators of cell–cell interactions given their outermost location on cell surfaces [28]. Pathogenic bacteria, including UPEC, can bind tissue‐ or cell‐specific glycoconjugates, supporting adhesion to host cells and playing a key role in determining pathogen tropism [29]. To resist urine flow and colonize the bladder, UPEC adhere to the urothelium via bacterial surface appendages, called pili or fimbriae [30]. Attachment to specific host glycans occurs through adhesins, a family of carbohydrate‐binding proteins located at the pili tip [30]. In addition to adhesion, pili‐mediated binding of surface glycans initiates UPEC invasion of urothelial cells via a zippering mechanism involving close associations between the bacteria and the host cell surface [31, 32]. Glycans also contribute to the process by which bacteria proliferate into large intracellular bacterial communities (IBCs) protected by insulating capsular polysaccharides [29, 30]. Host glycans present in the mucus layer, such as GAGs, and on the surface of secreted glycoproteins, such as THP, in turn, prevent bacterial attachment to the urothelium and promote bacterial elimination in urine [33, 34].

Here, we bring together experimental evidence describing how glycans mediate or prevent UPEC infection of the bladder, highlighting the relevance of glycan‐dependent interactions in UTI at different steps of the infection and immune response. Finally, we provide an updated view on the current state of sugar‐based treatments for UTI as a valuable alternative to antibiotics in the wake of disseminated multidrug resistance.

## CARBOHYDRATES DIRECTLY MEDIATE UPEC ATTACHMENT, INTERNALIZATION AND PROLIFERATION

UPEC interact with host glycans at very early stages of infection, in which the bacteria use filamentous adhesive organelles called type 1 pili to adhere to urothelial cells in the bladder [31, 32, 35, 36]. The coiled polymeric structure found in these pili terminates at its distal tip with the adhesin FimH, which mediates binding of UPEC to the urothelium [37, 38]. Type 1 pili or FimH mutants are unable to adhere to the urothelium in a mouse model of cystitis or invade bladder cells in vitro [32, 39]. FimH has two protein domains: the pilus‐linking pilin domain and the distal lectin domain, which binds carbohydrates [38, 40]. The FimH lectin domain binds terminal mannose(α1,3)‐mannose residues with high affinity and, to a lesser extent, mannose(α1,2)‐mannose and mannose (α1,6)‐mannose [41, 42]. These constitute the terminal moieties of high‐mannose N‐glycans, which are particularly abundant on UP1a and integrin α3β1 [36, 43, 44].

As UPs almost entirely cover the luminal surface of umbrella cells, UP1a mannose groups are the most frequently encountered FimH receptor on the urothelium of many species, including humans, mice and cattle [15, 36, 43]. Interestingly, the presence of N‐acetylglucosamine (GlcNAc), at the high‐mannose core, increases FimH affinity towards mannosides [41]. Molecular docking models predict that the mannose(β1‐4)‐GlcNAc motif stabilizes the terminal mannose insertion in the cavity of the FimH mannose‐binding pocket [41]. Considering that FimH preferentially binds terminally exposed mannoses [43], these results suggest that interactions with the N‐glycan core of high‐mannose glycans may stabilize FimH binding. Nevertheless, whether UPEC shows differential affinity towards terminal or core mannoses during UTI remains to be established.

**FIGURE 1:**
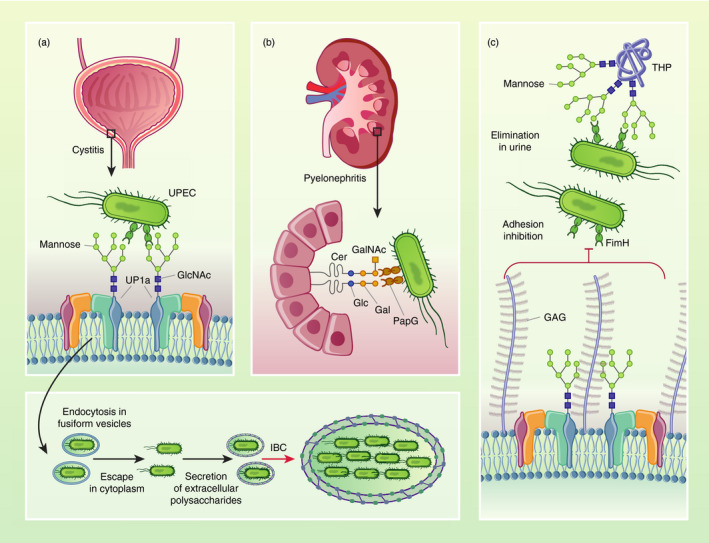
Glycan‐dependent interactions that promote or prevent infection in the urinary tract. Uropathogenic *E*.* coli* (UPEC) colonize the bladder by adhering to the urothelium via hair‐like extracellular appendages called pili, which have carbohydrate‐binding proteins at their tips. (a) The major facilitator of colonization is the adhesin FimH, which binds mannose moieties of high‐mannose structures on uroplakins (UPs) covering urothelial cell membranes. In the bladder, mannose‐specific FimH‐mediated UPEC binding induces bacterial internalization into umbrella cells via endocytosis into discoidal/fusiform vesicles. Internalized UPEC escape into the cytoplasm, where they aggregate and rapidly proliferate to form densely packed intracellular bacterial communities (IBCs). Extracellular bacterial polysaccharides, such as the K1 capsule, contribute to the formation, shape, and growth of IBCs. (b) UPEC can also ascend the ureters to the kidneys to cause pyelonephritis by invading and proliferating in renal tubular epithelial cells. To adhere to the kidney epithelium, UPEC typically use the galactose‐specific PapG adhesins on P pili, which bind galactose‐rich membrane glycolipids, including globotetraosylceramides and globotriaosylceramides. (c) Host glycans can prevent UPEC adhesion to the urothelium. Glycosaminoglycan (GAG)‐rich proteoglycans form a gel‐like mucus layer on the luminal surface of the bladder, which blocks FimH‐mediated binding to mannosylated glycoproteins on the urothelium, inhibiting invasion of urothelial cells. In the lumen, highly glycosylated Tamm–Horsfall protein (THP) has uroplakin‐like mannose glycans, which bind FimH with high affinity, preventing UPEC adhesion. Abbreviations: Cer – ceramide, Gal – galactose, GalNAc – *N*‐acetylgalactosamine, Glc – glucose, GlcNAc – *N*‐acetylglucosamine, Man – mannose

After binding, FimH minimizes the probability of UPEC expulsion during micturition by enhancing its mannose‐binding strength via a catch‐bond mechanism, whereby the binding pocket affinity for mannosylated structures increases under the tensile mechanical force exerted by urine flow [45, 46]. Given the prevalence of UPEC‐mediated UTI, the abundance of UP on the urothelium and the requirement of FimH‐mediated UPEC adhesion to initiate UTI, binding of FimH to mannose groups of UP1a exemplifies a key glycan target to disrupt host–pathogen interactions (Figure 1a).

Tissue glycosylation can vary over disease progression or following environmental changes, such as diet, smoking or age. It is therefore relevant to ask whether interindividual differences or long‐lasting changes in urothelial glycosylation may differentially contribute to the risk of UTI. Interestingly, galactosyltransferase expression is upregulated in mouse bladders in UTI, and UPEC increase the expression of Fml pili with its associated FimH‐like FmlH adhesin, which has high affinity for galactose‐containing epitopes in chronic UTI models [47]. Microscopic analysis with fluorescence‐tagged FmlH stains the GalNAc‐rich intermediate urothelial layer of mouse bladders only in chronically infected mice, whereas staining in naïve bladders is scattered [47]. As exfoliation of bladder apical cells exposes the underlying intermediate urothelium, this observation suggests that the FmlH adhesin may help UPEC to resist elimination in urine by binding to the intermediate urothelium surface later in infection.

Once UPEC access the bladder, FimH‐mediated binding to terminal mannoses on host UP or integrin α3β1 induces UPEC engulfment by umbrella cells [31, 32, 44, 48]. Cross‐linking of other FimH membrane receptors (reviewed elsewhere [30]) on adjacent lipid rafts activates downstream signalling pathways, such as Rho family GTPases, leading to actin cytoskeleton reorganization and UPEC endocytosis via intracellular trafficking proteins, such as dynamins [31, 32, 49]. The plasma membrane zippers around attached pili, facilitating the intimate binding of UPEC to the urothelium by engaging other coreceptors in lipid rafts [32, 44, 49, 50]. Upon entry, some UPEC escape into the cytoplasm and proliferate rapidly to form clonal IBCs, which are biofilm‐like, intracytoplasmic masses of bacteria that assume a coccoid shape [51, 52, 53, 54, 55]. A small number of UPEC may also invade intermediate cells and persist in late endosomal vesicles surrounded by a ‘cocoon’ of actin filaments, as quiescent intracellular reservoirs over periods of time [51, 53, 56].

UPEC induce host cell defence mechanisms arising from host recognition of pathogen‐associated molecular patterns, such as lipopolysaccharides, or other bacterial factors not yet identified [48, 57]. For example, FimH‐mediated bacterial adhesion to mannosylated sites on umbrella cell membranes induces caspase‐dependent apoptosis and exfoliation of these cells, leading to elimination of IBCs [31, 48]. Additionally, umbrella cells can expel invading bacteria before IBC formation. UPEC hijack the bladder cell trafficking machinery to enter via Rab11b/Rab27b^+^ cAMP‐responsive fusiform vesicles; however, binding of intracellular TLR4 in infected urothelial cells induces exocytosis of UPEC‐containing vacuoles via similar Rab27b^+^ cAMP‐dependent pathways, thus reducing the number of intracellularly proliferating bacteria [58, 59, 60].

Interestingly, UPEC require FimH not only for binding of mannosylated host surface proteins and cell invasion, but also for intracellular survival and proliferation into IBCs [39, 61]. In a mouse model of UTI, UPEC mutants that do not express type 1 pili during the intracellular phase are decreased in number and do not organize into IBCs compared with infection with the control parental strain, demonstrating that type 1 pili aid formation of IBCs [39]. FimH specifically facilitates IBC development as mutations in two positively selected residues of the FimH pilin domain are sufficient to reduce IBCs in mice compared with animals infected with the wild‐type isogenic UPEC strain [61]. Strikingly, the fitness reduction in UPEC mutants is specific to the urinary tract, as gut colonization is comparable to wild‐type strain, and is apparent only at later time‐points after initial intracellular colonization, when IBC development has already begun [61]. This is because positively selected residues of the pilin domain affecting the aggregation of intracellular UPEC into IBCs do not impair the mannose‐binding capacity of FimH required for UPEC adhesion or invasion [61].

UPEC not only recognize host glycans, but they also synthesize their own set of carbohydrates to promote intracellular proliferation into IBCs [30] (Figure 1a). To limit their detection and favour proliferation, internalized UPEC secrete a protective polysaccharide‐rich matrix that can incorporate host membrane‐derived UPs [52]. The bacterial polysaccharide layer is poorly immunogenic and, as such, hides immunogenic molecular patterns from host intracellular recognition receptors [62]. In this way, IBC development may delay immune responses, such as caspase‐3‐mediated exfoliation of umbrella cells or phagocytosis [31, 63]. As *E*.* coli* strains secrete multiple types of surface‐enveloping or capsular extracellular polysaccharides, identifying the exact biochemical composition of the polysaccharide‐rich matrix produced by intracellular UPEC at different time‐points during UTI, including during formation of IBCs, will be challenging [53, 62].

Most UPEC isolates produce capsular polysaccharides, such as the polysialic acid K1 capsule, and other negatively charged polysaccharides that may polymerize into biofilm‐like structures, such as colanic acid, β‐1,6‐N‐acetyl‐D‐glucosamine or cellulose [62]. Specifically, K1 capsule and associated sialic acid signalling mediate a crucial structural role during intracellular proliferation in UTI [63]. Many IBC‐forming UPEC strains express K1 polysaccharides in vivo, and deficiencies in K1 capsule synthesis or assembly, compromising capsule production, result in comparably reduced bacterial counts in mice 2 weeks post‐infection [63]. K1‐deficient UPEC numbers also decrease more quickly over time in mice than bacterial CFU of a cystitis patient‐derived UPEC strain [63]. Similar to the type 1 pili or FimH pilin domain mutants [53, 61], the numbers of intracellular K1 capsule‐deficient UPEC bacteria are decreased because they fail to aggregate into IBCs. In fact, K1 capsule‐deficient UPEC mutants further altered to upregulate sialic acid trafficking and metabolism display partially restored capacities of K1 capsule synthesis and IBC development compared with K1 capsule‐deficient single‐mutant strains, showing that secreted polymeric glycans facilitate UPEC proliferation into IBCs [63]. However, the exaggerated catabolism and dysregulated sensing of intracellular sialic acid in the UPEC double mutants used in this study may have decreased intracellular concentrations of sialic acids, such as N‐acetylneuraminic acid (Neu5Ac) or N‐acetylglucosamine (GlcNAc) [63], which would normally downregulate the OFF‐to‐ON phase variation switch that selectively induces type 1 pili expression in UPEC during UTI [64, 65, 66, 67]. Therefore, it needs to be verified whether the partial restoration of IBC development capacity in these mutant UPEC is directly dependent on capsular polysaccharide metabolism rather than increased surface expression of type 1 pili. Future studies of the secreted polysaccharides of UPEC should also include investigation of their potential signalling functions to elucidate whether these bacterial glycans inhibit intracellular immune response mechanisms.

## GLYCOLIPIDS SERVE AS SCAFFOLDS AND FOOTHOLDS FOR UPEC

Glycans are also attached to lipids on cell surfaces, creating a rich diversity of glycolipid structures. In the urothelium, glycolipids maintain the steep osmotic gradient between urine and plasma [15]. Biochemical and chromatographic studies of the bladder show that the luminal plasma membrane is made primarily of ceramides, which are glycosphingolipids consisting of a sphingosine backbone, one fatty acid tail and a polar head made of the monosaccharides D‐glucose or D‐galactose [68, 69]. Umbrella cell membranes have a non‐symmetrical ceramide distribution, with higher expression in the apical leaflet of the lipid bilayer compared with the cytoplasmic side [68]. In addition to reduced permeability compared with phospholipids, the height of the ceramides and of the embedded uroplakins is very similar, enhancing the structural stability of UP in umbrella cell membranes [70]. Although glycolipids constitute 62% of the plaque three‐dimensional structure, including the hollow centre with the FimH‐targeted high‐mannose structures [70], the bacterial factors governing UPEC interaction with bladder glycosphingolipids during UTI remain unknown.

Glycolipids play an important role when bacteria reach the kidneys, where UPEC preferentially bind to the galactose moieties of globotetraosylceramides (Gb4Cer) and globotriaosylceramides (Gb3Cer), two classes of ceramides bearing a combination of N‐acetylgalactosamine, D‐glucose, and D‐galactose, which are highly expressed in the kidneys [69, 71, 72]. To mediate binding, UPEC use the attachment appendages called P pili. These are heteropolymeric fibres capped by the PapG adhesion molecule, which has high affinity for galactose(αl‐4)galactose epitopes, such as those found in Gb4Cer and Gb3Cer [72, 73]. Thus, UPEC strains that express P pili can bind the kidney tubular papillary epithelium and cause pyelonephritis [73] (Figure 1b). Similar analyses in umbrella cells will help to determine whether UPEC require the binding of specific membrane glycolipids after FimH‐mediated adhesion to facilitate the engagement of lipid rafts initiating cell invasion.

## BLADDER GLYCOSAMINOGLYCANS PREVENT UPEC ADHESION

As an added layer of complexity in the glycobiology of the bladder barrier, the urothelium is physically separated from harmful metabolites or invading uropathogens by a protective layer of secreted or membrane‐tethered GAGs, as well as proteoglycans, which are linearized proteins decorated with a high number of different GAGs [24]. GAGs are long, negatively charged linear polysaccharides formed by polymerization of repeating disaccharide monomers made of amino sugars, such as sialic acids (N‐acetylneuraminic acid [Neu5Ac] in humans), and a galactose or an oxidized monosaccharide, such as D‐glucuronic acid [74]. Heparan sulphate (54%), chondroitin sulphate (29%) and dermatan sulphate (17%) are the most common GAGs in the human bladder [74]. In all cases, the long sugar chains in GAGs and proteoglycans give these molecules the capacity to strongly bind water molecules via intermolecular forces, thus trapping them into a gel‐like structure that separates the bladder urothelium from urine and non‐gaseous molecules.

GAG concentration in human urine samples or from pig bladder scrapings suggests that the mucus lining the bladder is thin and largely made of proteoglycans [75]. The bladder GAG layer is about 10 times thinner than the mucus in the mouse colon [76], supporting the idea that the bladder surface requires different protection compared with the intestine. Bladder tissue is not absorptive like the gut and instead prevents the entry of non‐gaseous material from the lumen through cell–cell junctional rings and surface UP [15, 68]. Beyond the intrinsically different functions and structures of the bladder and gut, the presence of different numbers and types of commensal bacteria or metabolites likely requires different barriers in the two mucosal tissues [15, 77]. Additionally, copious mucus secretion may pose an obstacle to urination.

However thin, the bladder mucus prevents bacterial adhesion to urothelium [78] (Figure 1c). Non‐specific or GAG‐specific chemically mediated depletion of the bladder mucus layer leads to increased attachment of radioactively labelled UPEC, *Klebsiella pneumoniae* or *Staphylococcus aureus* in a rabbit model, supporting that bladder mucus impedes adherence to the urothelium [78, 79, 80]. Interestingly, observation via scanning and transmission electron microscopy of bladder mucus in a rat model of UTI with UPEC shows a membrane‐bound layer of hair‐like GAGs entrapping large microcolonies of bacteria surrounded by a glycocalyx [81]. These micrographs support the idea that to reach mannosylated glycoproteins on umbrella cell surfaces with their pili, UPEC may adhere to and burrow through the mucus polymers. Although bladder mucus degradation by UPEC has never been formally demonstrated and no pathogenic K1 serotypes of extraintestinal *E*.* coli* express specific glycosaminolytic enzymes [82], UPEC may penetrate the GAG layer by secretion of serine protease autotransporters of *Enterobacteriaceae* (SPATE). Some SPATE, such as Pic, display dose‐dependent glycosaminolytic activity in vitro [83]. However, mice inoculated with a pyelonephritis patient‐derived UPEC strain or a Pic‐deficient mutant show similar bacterial burden in bladder and kidneys 6 days post‐infection, suggesting that Pic deficiency does not impair UPEC fitness in vivo [84].

Urinary tract microbiota may also influence UPEC fitness during infection by mucus consumption [77]. As microbiota‐liberated GAG carbohydrates regulate the growth of enterohaemorrhagic *E*.* coli* in the intestine, the relative proportion of mucus‐degrading bacteria species may favour UPEC infection by secretion of glycosaminolytic enzymes [85]. Oral administration of mucus‐constituting glycans to mice tempers the detrimental perturbations of the gut microbial community by favouring the growth of mucus‐consuming commensals that are prevalent in the healthy gut [86]. Given the differences between mucus layers of the gastrointestinal and urinary tract, it would be interesting to test whether modulating the relative abundance of urinary tract commensals feeding on bladder mucus impacts host susceptibility to UTI. Therefore, future studies should establish whether UPEC require the presence of a mucus‐degrading commensal population to bind urothelium, or whether SPATE or other unknown virulence factors permit UPEC to penetrate the host mucus to reach the urothelial surface.

## TAMM–HORSFALL PROTEIN, A CRITICAL HOST DEFENCE MOLECULE

In addition to GAGs, non‐glycosylated cationic antimicrobial peptides, such as β‐defensins or cathelicidin, contribute to protection of the bladder urothelium against uropathogens and toxins [27]. The most abundant glycoprotein in urine is Tamm–Horsfall protein (THP also called uromodulin), which is constitutively secreted in the kidneys, specifically by the renal cells in the thick ascending limb of the loop of Henle [34]. In urine, THP forms large multimeric aggregates with a flexible zigzag‐shaped backbone and protruding hair‐like filaments that physically prevent UPEC interaction with the urothelium by entrapping bacteria and clumping them together with its highly glycosylated surface [87]. Each THP monomer contains mannosylated and sialylated N‐glycans that may antagonize glycan–uropathogen interactions, acting in urine as a multivalent molecular ‘decoy’ [87]. The high‐mannose structures on the surface of THP strongly bind type 1‐piliated UPEC in a mannose‐dependent fashion, and THP deficiency results in greater bacterial numbers in mouse bladders, supporting that THP glycans are protective against UPEC infection in the bladder [88, 89, 90] (Figure 1c). Light microscopy or cryoelectron tomography analysis of urine from UTI patients infected with several uropathogens, including UPEC, reveals multiple bacterial aggregates associated with and surrounded by THP filaments, which even display clear contacts with pilus tips [87]. Consequently, changes in the glycosylation of THP may make the human urinary tract more permissive to infection. For instance, patients with type I diabetes have lower amounts of sialic acid in urinary THP compared with healthy volunteers, which may contribute to the higher risk of UTI among diabetic patients [91].

In addition to type 1 pili and P pili, which are associated with UPEC infection of the bladder and kidneys, respectively, another type of filamentous adherence structure, called S pilus, mediates binding in the urinary tract [34, 92]. Whereas type 1 and P pili recognize and bind mannose and galactose disaccharides, respectively, S pili specifically adhere to sialic acids capping terminal galactosides [NeuAc(α2,3)Gal], which are found in the branches or antennae of THP N‐glycans [34]. Consequently, THP may be protective against kidney infections by S‐piliated UPEC as renal luminal cells of the distal tubules are also enriched with surface oligosaccharides containing NeuAc(α2,3)Gal [34]. However, the protective role of THP against S‐piliated UPEC needs verification because the binding of THP by the adhesin of S pili has not been experimentally demonstrated, and the functional role of S pili during UTI remains unclear [92]. Interestingly, atomic force microscopy experiments with an S‐piliated UPEC strain reveal that the helix‐like structure of the S pili shaft has the most rapid relaxation kinetics compared with type 1 and P pili, indicating that when pili unravel to absorb shear stress forces, such as urine flow, S pili are the first to recover their initial quaternary structure [92]. A faster relaxation of the rod may reduce the load exerted on the apical adhesin, potentially extending the duration of adhesion to the sialic acid‐rich urothelium of the upper urinary tract, thereby favouring the ascension of S‐piliated UPEC to the kidneys. Therefore, future glycobiology studies are needed to test the putative protection provided by THP against infection with S‐piliated UPEC strains in vivo, as well as determine binding properties and the role of the S pilus during bladder and kidney infections.

Infected mice lacking THP have not only increased bacterial counts in the urine and bladder, but also elevated mortality that may be due to an exaggerated inflammatory response [89, 90]. Indeed, significantly increased neutrophil numbers are present in the urine of THP‐null mice 24 h after instillation of PBS into the bladder [93]. Greater neutrophil infiltration may be attributable to THP glycans as the surface cell protein sialic acid‐binding immunoglobulin‐type lectin‐9 (Siglec‐9, Siglec‐E in mice) in human neutrophils directly binds to the structure Neu5Ac(α2,3)Gal(β1,4)GlcNAc on THP to decrease reactive oxygen species ex vivo [93]. These studies provide evidence to suggest that THP glycans not only directly protect against UTI as decoys for bacterial receptors, but may also regulate immunity to UTI by blunting the activation of infiltrating immune cells, such as neutrophils, where an exaggerated antimicrobial response may impair urothelial barrier integrity. Nonetheless, whether inhibition of neutrophil oxidative burst via THP‐mediated engagement of Siglec‐9/Siglec‐E receptors has consequences on UTI needs to be verified in vivo. Altogether, the above studies highlight how integral THP is to bladder immunity and support a renewed translational research interest in THP glycosylation to explore its underappreciated barrier and immunomodulatory roles for clinical applications.

## TRANSLATIONAL GLYCOBIOLOGY IN UTI: HOW TO SWEET TALK UPEC

More than 20 years ago, the finding that a FimH null mutant of UPEC had impaired colonization and induced less inflammation in mouse bladders, together with the widespread expression of FimH in UPEC strains, supported the idea that a FimH‐targeted vaccine may provide positive therapeutic outcomes [35, 94, 95]. While this idea has not entirely borne out, as these vaccines have only shown preclinical efficacy [95], understanding the glycobiology of UPEC infection and immunity may offer additional opportunities to target FimH and other glycan‐dependent interactions in the clinic [96, 97]. Administration of a mixture of selective sugar antagonists for UPEC adhesins via a catheter may be an inexpensive strategy to significantly decrease the risk of developing UTI [98]. By passively targeting invading uropathogens, sugar‐derived drugs or glycomimetics have the potential to treat UTI and prevent recurrence regardless of most host‐dependent factors.

As the mannose‐specific recognition of FimH adhesin is well established, and nearly all UPEC strains express type 1 pili, many glycobiology studies focus on blocking this crucial interaction [41, 96, 99]. The earliest examples are soluble monosaccharides, such as D‐mannose, which block lectin‐mediated adhesion of uropathogens to urothelial cells in vitro [100]. Promisingly, daily oral intake of D‐mannose supplements has the same efficacy as the antibiotic nitrofurantoin in reducing the frequency of infection in women with recurrent UTI [101]. Addition of D‐mannose to UPEC incubated with human THP did not alter UPEC association with THP filaments or THP‐mediated UPEC clumping in vitro [87], suggesting that prophylactic administration of D‐mannose to patients with recurrent UTI may synergize with urinary THP to antagonize UPEC adhesion. To further test its therapeutic potential, an ongoing double‐blinded placebo‐controlled clinical trial in the UK has recruited 598 women to test whether daily intake of D‐mannose reduces the rate of UTI recurrence within a 6‐month period [102].

Aromatic alpha‐mannosides, molecules containing a mannose linked to a benzene group, are a promising alternative to D‐mannose due to their comparatively increased affinity for the mannose‐binding domain of FimH [96]. Rationally designed, high‐affinity mannoside antagonists of FimH block UPEC adhesion to cognate urothelial carbohydrates, preventing UPEC invasion [97, 98]. Oral treatment with the high‐affinity FimH antagonist mannoside M4284 reduces UPEC burden in the colon, bladder, and kidneys of mice intentionally colonized with UPEC in both the gut and urinary tract, compared with D‐mannose control treatment [103]. This study demonstrates that mannosides not only treat UTI but may reduce the risk of new episodes by depleting tissue‐associated UPEC reservoirs. Oral administration of a mannoside‐containing compound to mice shortly before and after UPEC instillation, respectively mimicking prophylaxis and treatment, significantly reduces early colonization levels in the bladder, demonstrating that sugar‐derived drugs have the potential to treat UTI [98]. Mannoside antagonists further reduce bacterial numbers in mouse bladders when administered in combination with a standard antibiotic regimen for UTI compared with either treatment alone, suggesting that sugar‐based drugs may enhance bacterial killing by UPEC sequestration in urine. Therefore, glycan‐based drugs could reduce the use of antibiotics, which will be of utmost importance as UPEC strains exhibit diverse and widespread antibiotic resistance [5].

Based on promising experimental and clinical evidence collected so far, sugar‐based mannose‐specific FimH antagonists may be a safe and inexpensive drug to treat cystitis. Although their efficacy may not be as potent as antibiotics, carbohydrate‐based anti‐adhesives are not expected to select for resistance genes or have detrimental side effects on the microbiota, thus arguing in favour of their use for treatment of UTI and prophylaxis against recurrence [104]. Overall, promoting the prophylactic use of effective antimicrobial glycan‐based therapeutics as an alternative to antibiotics, as well as determining the consequences of their long‐term use, could be an efficient approach to limit the impact of antimicrobial resistance.

Learning from the successful clinical translation of mannose‐based therapeutics for treatment of cystitis, future glycobiology studies may consider whether galactose‐rich bioactive compounds that target PapG adhesins and are retained in the kidneys could inhibit the severity of pyelonephritis. For translational studies, some caution should be taken when using animal models. Basic glycosylation pathways are conserved in mammals, but some differences can be found between humans and common laboratory species. Understanding these structural differences in GAGs, glycoproteins, and glycolipids would facilitate the design of glycan‐based drugs. Glycan structural complexity and diversity are major obstacles for the translation of basic discoveries to glycan‐based therapeutics. Structural analysis relies on mass spectrometry or nuclear magnetic resonance spectroscopy, which are time‐consuming, expensive and require large amount of biological material. However, recent breakthroughs in the field [105], such as novel arraying methods and improved carbohydrate synthesis, may foster translational glycobiology in the next years.

## FINAL REMARKS

In conclusion, carbohydrates play a prominent role in host invasion and response during UTI. Following UPEC infection, FimH‐mediated mannose binding induces adhesion and invasion of the urothelium, which is aided by bacterial capsular polysaccharides. However, host GAGs and proteoglycans protect the urothelium from bacterial adhesion, thereby reducing bacterial invasion. In addition, secreted, highly glycosylated glycoproteins, such as THP, inhibit UPEC attachment by binding to FimH or other bacterial adhesion proteins to reduce bacterial burden. Lectin antagonists show encouraging results in the treatment of UTI, revealing a promising horizon for translational glycobiology and supporting that the investigation of glycans involved in UTI will drive the discovery of targets for non‐antibiotic therapies based on sugars.

## CONFLICT OF INTEREST

The authors declare that no conflict of interest exists.

## References

[1] Ozturk R, Murt A. Epidemiology of urological infections: a global burden. World J Urol. 2020;38:2669–79. 10.1007/s00345-019-03071-4 31925549

[2] Foxman B, Brown P. Epidemiology of urinary tract infections ‐ Transmission and risk factors, incidence, and costs. Infect Dis Clin North Am. 2003;17:227–41. 10.1016/S0891-5520(03)00005-9 12848468

[3] Ingersoll MA. Sex differences shape the response to infectious diseases. PLOS Pathog. 2017;13:e1006688. 10.1371/journal.ppat.1006688 29284060 PMC5746274

[4] Scharff AZ, Rousseau M, Mariano LL, Canton T, Consiglio CR, Albert ML, et al. Sex differences in IL‐17 contribute to chronicity in male versus female urinary tract infection. JCI insight. 2019;2019:5. 10.1172/jci.insight.122998 PMC6629110 31145099

[5] Flores‐Mireles AL, Walker JN, Caparon M, Hultgren SJ. Urinary tract infections: epidemiology, mechanisms of infection and treatment options. Nat Rev Microbiol 2015;13:269–84. 10.1038/nrmicro3432 25853778 PMC4457377

[6] Foxman B. The epidemiology of urinary tract infection. Review. Nat Rev Urol. 2010;7:653–60. 10.1038/nrurol.2010.190 21139641

[7] Gorczynska E, Turkiewicz D, Rybka K, Toporski J, Kalwak K, Dyla A, et al. Incidence, clinical outcome, and management of virus‐induced hemorrhagic cystitis in children and adolescents after allogeneic hematopoietic cell transplantation. Biol Blood Marrow Transplant. 2005;11:797–804. 10.1016/j.bbmt.2005.06.007 16182180

[8] Shapiro T, Dalton M, Hammock J, Lavery R, Matjucha J, Salo DF. The prevalence of urinary tract infections and sexually transmitted disease in women with symptoms of a simple urinary tract infection stratified by low colony count criteria. Acad Emerg Med. 2005;12:38–44. 10.1197/j.aem.2004.08.051 15635136

[9] Moore EE, Hawes SE, Scholes D, Boyko EJ, Hughes JP, Fihn SD. Sexual intercourse and risk of symptomatic urinary tract infection in post‐menopausal women. J Gen Intern Med. 2008;23:595–9. 10.1007/s11606-008-0535-y 18266044 PMC2324148

[10] Magruder M, Sholi AN, Gong C, Zhang L, Edusei E, Huang J, et al. Gut uropathogen abundance is a risk factor for development of bacteriuria and urinary tract infection. Nat Commun. 2019;10:5521. 10.1038/s41467-019-13467-w 31797927 PMC6893017

[11] Dill‐McFarland KA, Tang Z‐Z, Kemis JH, Kerby RL, Chen G, Palloni A, et al. Close social relationships correlate with human gut microbiota composition. Sci Rep. 2019;9:9703. 10.1038/s41598-018-37298-9 PMC6345772 30679677

[12] Vihta K‐D, Stoesser N, Llewelyn MJ, Quan TP, Davies T, Fawcett NJ, et al. Trends over time in *Escherichia coli* bloodstream infections, urinary tract infections, and antibiotic susceptibilities in Oxfordshire, UK, 1998–2016: a study of electronic health records. Lancet Infect Dis. 2018;18:1138–49. 10.1016/s1473-3099(18)30353-0 30126643 PMC7612540

[13] Lipsky BA. Prostatitis and urinary tract infection in men: what's new; What's true? Am J Med. 1999;106:327–34. 10.1016/s0002-9343(99)00017-0 10190383

[14] Lupo F, Ingersoll MA. Is bacterial prostatitis a urinary tract infection? Nat Rev Urol. 2019;16:203–4. 10.1038/s41585-019-0150-1 30700862

[15] Jost SP, Gosling JA, Dixon JS. The morphology of normal human bladder urothelium. J Anat. 1989;167:103–15. 2630525 PMC1256824

[16] Mathai JC, Zhou EH, Yu W, Kim JH, Zhou G, Liao Y, et al. Hypercompliant apical membranes of bladder umbrella cells. Biophys J. 2014;107:1273–9. 10.1016/j.bpj.2014.07.047 25229135 PMC4167298

[17] Wu X‐R, Kong X‐P, Pellicer A, Kreibich G, Sun T‐T. Uroplakins in urothelial biology, function, and disease. Kidney Int. 2009;75:1153–65. 10.1038/ki.2009.73 19340092 PMC3717210

[18] Katnik‐Prastowska I, Lis J, Matejuk A. Glycosylation of uroplakins. Implications for bladder physiopathology. Glycoconj J. 2014;31:623–36. 10.1007/s10719-014-9564-4 25394961 PMC4245495

[19] Khandelwal P, Ruiz WG, Balestreire‐Hawryluk E, Weisz OA, Goldenring JR, Apodaca G. Rab11a‐dependent exocytosis of discoidal/fusiform vesicles in bladder umbrella cells. Proc Natl Acad Sci USA. 2008;105:15773–8. 10.1073/pnas.0805636105 18843107 PMC2572972

[20] Khandelwal P, Ruiz WG, Apodaca G. Compensatory endocytosis in bladder umbrella cells occurs through an integrin‐regulated and RhoA‐ and dynamin‐dependent pathway. Embo J. 2010;29:1961–75. 10.1038/emboj.2010.91 20461056 PMC2892371

[21] Khandelwal P, Prakasam HS, Clayton DR, Ruiz WG, Gallo LI, van Roekel D, et al. A Rab11a‐Rab8a‐Myo5B network promotes stretch‐regulated exocytosis in bladder umbrella cells. Mol Biol Cell. 2013;24:1007–19. 10.1091/mbc.E12-08-0568 23389633 PMC3608489

[22] Gallo LI, Dalghi MG, Clayton DR, Ruiz WG, Khandelwal P, Apodaca G. RAB27B requirement for stretch‐induced exocytosis in bladder umbrella cells. Am J Physiol Cell Physiol. 2018;314:C349–65. 10.1152/ajpcell.00218.2017 29167152 PMC6335015

[23] Eaton AF, Clayton DR, Ruiz WG, Griffiths SE, Rubio ME, Apodaca G. Expansion and contraction of the umbrella cell apical junctional ring in response to bladder filling and voiding. Mol Biol Cell. 2019;30:2037–52. 10.1091/mbc.E19-02-0115 31166831 PMC6727774

[24] Lilly JD, Parsons CL. Bladder surface glycosaminoglycans is a human epithelial permeability barrier. Surg Gynecol Obstet. 1990;171:493–6. 2244283

[25] Svanborgeden C, Svennerholm AM. Secretory immunoglobulin‐A and immunoglobulin‐G antibodies prevent adhesion of *Escherichia‐coli* to human urinary‐tract epithelial‐cells. Infect Immun. 1978;22:790–7. 83303 10.1128/iai.22.3.790-797.1978 PMC422230

[26] Zasloff M. Antimicrobial peptides, normally sterile urinary innate immunity, and the tract. J Am Soc Nephrol. 2007;18:2810–6. 10.1681/asn.2007050611 17942949

[27] Lacerda Mariano L, Ingersoll MA. The immune response to infection in the bladder. Nat Rev Urol. 2020; 17:439–58. 10.1038/s41585-020-0350-8 32661333

[28] Schnaar RL. Glycobiology simplified: diverse roles of glycan recognition in inflammation. J Leukoc Biol. 2016;99:825–38. 10.1189/jlb.3RI0116-021R 27004978 PMC4952015

[29] Poole J, Day CJ, von Itzstein M, Paton JC, Jennings MP. Glycointeractions in bacterial pathogenesis. Nat Rev Microbiol. 2018;16:440–52. 10.1038/s41579-018-0007-2 29674747

[30] Lewis AJ, Richards AC, Mulvey MA. Invasion of host cells and tissues by uropathogenic bacteria. Microbiol Spectrum 2016;4:0026–2016. 10.1128/microbiolspec.UTI-0026-2016 PMC5244466 28087946

[31] Mulvey MA, Lopez‐Boado YS, Wilson CL, Roth R, Parks WC, Heuser J, et al. Induction and evasion of host defenses by type 1‐piliated uropathogenic *Escherichia coli* . Science 1998;282:1494–7. 10.1126/science.282.5393.1494 9822381

[32] Martinez JJ, Mulvey MA, Schilling JD, Pinkner JS, Hultgren SJ. Type 1 pilus‐mediated bacterial invasion of bladder epithelial cells. Embo J. 2000;19:2803–12. 10.1093/emboj/19.12.2803 10856226 PMC203355

[33] Hurst RE, Zebrowski R. Identification of proteoglycans present at high‐density on bovine and human bladder luminal surface. J Urol. 1994;152:1641–5. 10.1016/s0022-5347(17)32495-3 7933221

[34] Serafini‐Cessi F, Monti A, Cavallone D. N‐Glycans carried by Tamm‐Horsfall glycoprotein have a crucial role in the defense against urinary tract diseases. Glycoconjugate J. 2005;22:383–94. 10.1007/s10719-005-2142-z 16622944

[35] Connell H, Agace W, Klemm P, Schembri M, Marild S, Svanborg C. Type 1 fimbrial expression enhances *Escherichia coli* virulence for the urinary tract. Proc Natl Acad Sci USA. 1996;93:9827–32. 10.1073/pnas.93.18.9827 8790416 PMC38514

[36] Zhou G, Mo WJ, Sebbel P, Min G, Neubert TA, Glockshuber R, et al. Uroplakin Ia is the urothelial receptor for uropathogenic *Escherichia coli*: evidence from in vitro FimH binding. J Cell Sci. 2001;114:4095–103. 11739641 10.1242/jcs.114.22.4095

[37] Jones CH, Pinkner JS, Roth R, Heuser J, Nicholes AV, Abraham SN, et al. FimH adhesin of type 1 pili is assembled into a fibrillar tip structure in the Enterobacteriaceae. Proc Natl Acad Sci USA. 1995;92:2081–5. 10.1073/pnas.92.6.2081 7892228 PMC42427

[38] Choudhury D, Thompson A, Stojanoff V, Langerman S, Pinkner J, Hultgren SJ. X‐ray structure of the FimC‐FimH chaperone‐adhesin complex from uropathogenic *Escherichia coli* . Science 1999;285:1061–6. 10.1126/science.285.5430.1061 10446051

[39] Wright KJ, Seed PC, Hultgren SJ. Development of intracellular bacterial communities of uropathogenic *Escherichia coli* depends on type 1 pili. Cel Microbiol. 2007;9:2230–41. 10.1111/j.1462-5822.2007.00952.x 17490405

[40] Schembri MA, Sokurenko EV, Klemm P. Functional flexibility of the FimH adhesin: insights from a random mutant library. Infect Immun. 2000;68:2638–46. 10.1128/iai.68.5.2638-2646.2000 10768955 PMC97470

[41] Bouckaert J, Mackenzie J, de Paz JL, Chipwaza B, Choudhury D, Zavialov A, et al. The affinity of the FimH fimbrial adhesin is receptor‐driven and quasi‐independent of *Escherichia coli* pathotypes. Mol Microbiol. 2006;61:1556–68. 10.1111/j.1365-2958.2006.05352.x 16930149 PMC1618777

[42] Dumych T, Bridot C, Gouin SG, Lensink M, Paryzhak S, Szunerits S, et al. A novel integrated way for deciphering the glycan code for the FimH Lectin. Molecules 2018;23:2794. 10.3390/molecules23112794 PMC6278545 30373288

[43] Xie B, Zhou G, Chan S‐Y, Shapiro E, Kong X‐P, Wu X‐R, et al. Distinct glycan structures of uroplakins Ia and Ib ‐ Structural basis for the selective binding of FimH adhesin to uroplakin Ia. J Biol Chem. 2006;281:14644–53. 10.1074/jbc.M600877200 16567801

[44] Eto DS, Jones TA, Sundsbak JL, Mulvey MA. Integrin‐mediated host cell invasion by type 1–piliated uropathogenic *Escherichia coli* . PLoS Pathog. 2007;3:949–61. 10.1371/journal.ppat.0030100 PMC1914067 17630833

[45] Aprikian P, Tchesnokova V, Kidd B, Yakovenko O, Yarov‐Yarovoy V, Trinchina E, et al. Interdomain interaction in the FimH adhesin of *Escherichia coli* regulates the affinity to mannose. J Biol Chem. 2007;282:23437–46. 10.1074/jbc.M702037200 17567583

[46] Sauer MM, Jakob RP, Eras J, Baday S, Eriş D, Navarra G, et al. Catch‐bond mechanism of the bacterial adhesin FimH. Nat Commun. 2016;7:710738. 10.1038/ncomms10738 PMC4786642 26948702

[47] Conover MS, Ruer S, Taganna J, Kalas V, De Greve H, Pinkner JS, et al. Inflammation‐induced adhesin‐receptor interaction provides a fitness advantage to uropathogenic *E‐coli* during chronic infection. Cell Host Microbe. 2016;20:482–92. 10.1016/j.chom.2016.08.013 27667696 PMC5294914

[48] Mulvey MA, Schilling JD, Martinez JJ, Hultgren SJ. Bad bugs and beleaguered bladders: interplay between uropathogenic *Escherichia coli* and innate host defenses. Proc Natl Acad Sci USA. 2000;97:8829–35. 10.1073/pnas.97.16.8829 10922042 PMC34019

[49] Eto DS, Gordon HB, Dhakal BK, Jones TA, Mulvey MA. Clathrin, AP‐2, and the NPXY‐binding subset of alternate endocytic adaptors facilitate FimH‐mediated bacterial invasion of host cells. Cell Microbiol. 2008;10:2553–67. 10.1111/j.1462-5822.2008.01229.x 18754852

[50] Wang H, Liang F‐X, Kong X‐P. Characteristics of the phagocytic cup induced by uropathogenic *Escherichia coli* . J Histochem Cytochem. 2008;56:597–604. 10.1369/jhc.2008.950923 18347076 PMC2386762

[51] Mulvey MA, Schilling JD, Hultgren SJ. Establishment of a persistent *Escherichia coli* reservoir during the acute phase of a bladder infection. Infect Immun. 2001;69:4572–9. 10.1128/iai.69.7.4572-4579.2001 11402001 PMC98534

[52] Anderson GG, Palermo JJ, Schilling JD, Roth R, Heuser J, Hultgren SJ. Intracellular bacterial biofilm‐like pods in urinary tract infections. Science 2003;301:105–7. 10.1126/science.1084550 12843396

[53] Justice SS, Hung C, Theriot JA, Fletcher DA, Anderson GG, Footer MJ, et al. Differentiation and developmental pathways of uropathogenic *Escherichia coli* in urinary tract pathogenesis. Proc Natl Acad Sci USA. 2004;101:1333–8. 10.1073/pnas.0308125100 14739341 PMC337053

[54] Rosen DA, Hooton TM, Stamm WE, Humphrey PA, Hultgren SJ. Detection of intracellular bacterial communities in human urinary tract infection. PLoS Med. 2007;4:1949–58. 10.1371/journal.pmed.0040329 PMC2140087 18092884

[55] Schwartz DJ, Chen SL, Hultgren SJ, Seed PC. Population dynamics and niche distribution of uropathogenic *Escherichia coli* during acute and chronic urinary tract infection. Infect Immun. 2011;79:4250–9. 10.1128/iai.05339-11 21807904 PMC3187256

[56] Mysorekar IU, Hultgren SJ. Mechanisms of uropathogenic *Escherichia coli* persistence and eradication from the urinary tract. Proc Natl Acad Sci USA. 2006;103:14170–5. 10.1073/pnas.0602136103 16968784 PMC1564066

[57] Schilling JD, Martin SM, Hunstad DA, Patel KP, Mulvey MA, Justice SS, et al. CD14‐ and Toll‐like receptor‐dependent activation of bladder epithelial cells by lipopolysaccharide and type 1 piliated *Escherichia coli* . Infect Immun. 2003;71:1470–80. 10.1128/iai.71.3.1470-1480.2003 12595465 PMC148872

[58] Bishop BL, Duncan MJ, Song J, Li G, Zaas D, Abraham SN. Cyclic AMP‐regulated exocytosis of *Escherichia coli* from infected bladder epithelial cells. Nat Med. 2007;13:625–30. 10.1038/nm1572 17417648

[59] Song J, Bishop BL, Li G, Grady R, Stapleton A, Abraham SN. TLR4‐mediated expulsion of bacteria from infected bladder epithelial cells. Proc Natl Acad Sci USA. 2009;106:14966–71. 10.1073/pnas.0900527106 19706440 PMC2736405

[60] Miao Y, Bist P, Wu J, Zhao Q, Li Q‐J, Wan Y, et al. Collaboration between distinct Rab Small GTPase trafficking circuits mediates bacterial clearance from the bladder epithelium. Cell Host Microbe. 2017;22:330–42.e4. 10.1016/j.chom.2017.08.002 28910634 PMC5659305

[61] Chen SL, Hung CS, Pinkner JS, Walker JN, Cusumano CK, Li Z, et al. Positive selection identifies an in vivo role for FimH during urinary tract infection in addition to mannose binding. Proc Natl Acad Sci USA. 2009;106:22439–44. 10.1073/pnas.0902179106 20018753 PMC2794649

[62] Whitfield C. Biosynthesis and assembly of capsular polysaccharides in *Escherichia coli* . Annu Rev Biochem. 2006;2006:39–68. 10.1146/annurev.biochem.75.103004.142545 16756484

[63] Anderson GG, Goller CC, Justice S, Hultgren SJ, Seed PC. Polysaccharide capsule and sialic acid‐mediated regulation promote biofilm‐like intracellular bacterial communities during cystitis. Infect Immun. 2010;78:963–75. 10.1128/iai.00925-09 20086090 PMC2825929

[64] Lim JK, Gunther NW, Zhao H, Johnson DE, Keay SK, Mobley HLT. In vivo phase variation of *Escherichia coli* type 1 fimbrial genes in women with urinary tract infection. Infect Immun. 1998;66:3303–10. 10.1128/iai.66.7.3303-3310.1998 9632599 PMC108346

[65] El‐Labany S, Sohanpal BK, Lahooti M, Akerman R, Blomfield IC. Distant cis‐active sequences and sialic acid control the expression of fimB in *Escherichia coli* K‐12. Mol Microbiol. 2003;49:1109–18. 10.1046/j.1365-2958.2003.03624.x 12890032

[66] Sohanpal BK, El‐Labany S, Lahooti M, Plumbridge JA, Blomfield IC. Integrated regulatory responses of fimB to N‐acetylneuraminic (sialic) acid and GlcNAc in *Escherichia coli* K‐12. Proc Natl Acad Sci USA. 2004;101:16322–7. 10.1073/pnas.0405821101 15534208 PMC526197

[67] Sohanpal BK, Friar S, Roobol J, Plumbridge JA, Blomfield IC. Multiple co‐regulatory elements and IHF are necessary for the control of fimB expression in response to sialic acid and N‐acetylglucosamine in *Escherichia coli* K‐12. Mol Microbiol. 2007;63:1223–36. 10.1111/j.1365-2958.2006.05583.x 17238917

[68] Hicks RM, Ketterer B, Warren RC. Ultrastructure and chemistry of luminal plasma‐membrane of mammalian urinary‐bladder ‐ structure with low permeability to water and ions. Philos Trans R Soc Lond B Biol Sci. 1974;268:23. 10.1098/rstb.1974.0013 4155088

[69] Lingwood CA. Glycosphingolipid functions. Cold Spring Harbor Perspect Biol. 2011;3:a004788. 10.1101/cshperspect.a004788 PMC3119914 21555406

[70] Min GW, Zhou G, Schapira M, Sun TT, Kong XP. Structural basis of urothelial permeability barrier function as revealed by Cryo‐EM studies of the 16 nm uroplakin particle. J Cell Sci. 2003;116:4087–94. 10.1242/jcs.00811 12972502

[71] Stapleton AE, Stroud MR, Hakomori SI, Stamm WE. The globoseries glycosphingolipid sialosyl galactosyl globoside is found in urinary tract tissues and is a preferred binding receptor in vitro for uropathogenic *Escherichia coli* expressing pap‐encoded adhesins. Infect Immun. 1998;66:3856–61. 10.1128/iai.66.8.3856-3861.1998 9673272 PMC108435

[72] Legros N, Ptascheck S, Pohlentz G, Karch H, Dobrindt U, Muething J. PapG subtype‐specific binding characteristics of *Escherichia coli* towards globo‐series glycosphingolipids of human kidney and bladder uroepithelial cells. Glycobiology 2019;29:789–802. 10.1093/glycob/cwz059 31361021

[73] Roberts JA, Marklund BI, Ilver D, Haslam D, Kaack MB, Baskin G et al. The Gal(alpha‐1‐4)Gal‐specific tip adhesin of *Escherichia‐coli* P‐fimbriae is needed for pyelonephritis to occur in the normal urinary. Proc Natl Acad Sci USA. 1994;91:11889–93. 10.1073/pnas.91.25.11889 7991552 PMC45341

[74] Hurst RE. Structure, function, and pathology of proteoglycans and glycosaminoglycans in the urinary‐tract. World J Urol. 1994;12:3–10. 8012413 10.1007/BF00182044

[75] N'Dow J, Jordan N, Robson CN, Neal DE, Pearson JP. The bladder does not appear to have a dynamic secreted continuous mucous gel layer. Research Support, Non‐U.S. Gov't. J Urol. 2005;173:2025‐31. 15879814 10.1097/01.ju.0000158454.47299.ae

[76] Johansson MEV, Phillipson M, Petersson J, Velcich A, Holm L, Hansson GC. The inner of the two Muc2 mucin‐dependent mucus layers in colon is devoid of bacteria. Proc Natl Acad Sci USA. 2008; 105:15064–9. 10.1073/pnas.0803124105 18806221 PMC2567493

[77] Brubaker L, Wolfe AJ. The female urinary microbiota, urinary health and common urinary disorders. Ann Transl Med. 2017;5:34. 10.21037/atm.2016.11.62 28217699 PMC5300856

[78] Parsons CL, Mulholland SG. Bladder surface mucin ‐ its antibacterial effect against various bacterial species. Am J Pathol. 1978;93:423. 362941 PMC2018387

[79] Parsons CL, Greenspan C, Moore SW, Mulholland S. Role of surface mucin in primary antibacterial defense of bladder. Urology 1977;9:48–52. 10.1016/0090-4295(77)90284-9 831354

[80] Parsons CL, Stauffer CW, Schmidt JD. Reversible inactivation of bladder surface glycosaminoglycan antibacterial activity by protamine sulfate. Infec Immun. 1988;56:1341–3. 10.1128/iai.56.5.1341-1343.1988 3281908 PMC259825

[81] Cornish J, Lecamwasam JP, Harrison G, Vanderwee MA, Miller TE. Host defense‐mechanisms in the bladder.2. Disruption of the layer of mucus. Br J Exp Pathol. 1988;69:759–70. 3064799 PMC2013295

[82] Severi E, Hood DW, Thomas GH. Sialic acid utilization by bacterial pathogens. Microbiology 2007;153:2817–22. 10.1099/mic.0.2007/009480-0 17768226

[83] Gutierrez‐Jimenez J, Arciniega I, Navarro‐Garcia F. The serine protease motif of Pic mediates a dose‐dependent mucolytic activity after binding to sugar constituents of the mucin substrate. Microb Pathog. 2008;45:115–23. 10.1016/j.micpath.2008.04.006 18538533

[84] Heimer SR, Rasko DA, Lockatell CV, Johnson DE, Mobley HLT. Autotransporter genes pic and tsh are associated with *Escherichia coli* strains that cause acute pyelonephritis and are expressed during urinary tract infection. Infect Immun. 2004;72:593–7. 10.1128/iai.72.1.593-597.2004 14688142 PMC343984

[85] Pacheco AR, Curtis MM, Ritchie JM, Munera D, Waldor MK, Moreira CG, et al. Fucose sensing regulates bacterial intestinal colonization. Nature 2012;492:113–7. 10.1038/nature11623 23160491 PMC3518558

[86] Pruss KM, Marcobal A, Southwick AM, Dahan D, Smits SA, Ferreyra JA, et al. Mucin‐derived O‐glycans supplemented to diet mitigate diverse microbiota perturbations. ISME J. 2021;15:577–91. 10.1038/s41396-020-00798-6 33087860 PMC8027378

[87] Weiss GL, Stanisich JJ, Sauer MM, Lin C‐W, Eras J, Zyla DS,, et al. Architecture and function of human uromodulin filaments in urinary tract infections. Science 2020;369:1005–10. 10.1126/science.aaz9866 32616672

[88] Reinhart HH, Obedeanu N, Sobel JD. Quantitation of Tamm‐Horsfall protein‐binding to uropathogenic *Escherichia‐coli* and lectins. J Infect Dis. 1990;162:1335–40. 10.1093/infdis/162.6.1335 1977810

[89] Mo L, Zhu XH, Huang HY, Shapiro E, Hasty DL, Wu XR. Ablation of the Tamm‐Horsfall protein gene increases susceptibility of mice to bladder colonization by type 1‐fimbriated *Escherichia coli* . Am J Physiol Renal Physiol. 2004;286:F795–802. 10.1152/ajprenal.00357.2003 14665435

[90] Bates JM, Raffi HM, Prasadan K, Mascarenhas R, Laszik Z, Maeda N, et al. Tamm‐Horsfall protein knockout mice are more prone to urinary tract infection. Kidney Int. 2004;65:791–7. 10.1111/j.1523-1755.2004.00452.x 14871399

[91] Rambausek M, Dulawa J, Jann K, Ritz E. Tamm Horsfall glycoprotein in diabetes mellitus: abnormal chemical composition and colloid stability. Eur J Clin Investig. 1988;18:237–42. 10.1111/j.1365-2362.1988.tb01252.x 3138126

[92] Castelain M, Sjostrom AE, Fallman E, Uhlin BE, Andersson M. Unfolding and refolding properties of S pili on extraintestinal pathogenic *Escherichia coli* . Eur Biophys J. 2010;39:1105–15. 10.1007/s00249-009-0552-8 19885656

[93] Patras KA, Coady A, Olson J, Ali SR, RamachandraRao SP, Kumar S, et al. Tamm‐Horsfall glycoprotein engages human Siglec‐9 to modulate neutrophil activation in the urinary tract. Immunol Cell Biology. 2017;95:960–5. 10.1038/icb.2017.63 PMC5698129 28829050

[94] Langermann S, Palaszynski S, Barnhart M, et al. Prevention of mucosal *Escherichia coli* infection by FimH‐adhesin‐based systemic vaccination. Science 1997; 276:607–11. 10.1126/science.276.5312.607 9110982

[95] Aziminia N, Hadjipavlou M, Philippou Y, Pandian SS, Malde S, Hammadeh MY. Vaccines for the prevention of recurrent urinary tract infections: a systematic review. BJU Int. 2019;123:753–68. 10.1111/bju.14606 30378242

[96] Bouckaert J, Berglund J, Schembri M, De Genst E, Cools L, Wuhrer M, et al. Receptor binding studies disclose a novel class of high‐affinity inhibitors of the *Escherichia coli* FimH adhesin. Mol Microbiol. 2005;55:441–55. 10.1111/j.1365-2958.2004.04415.x 15659162

[97] Wellens A, Garofalo C, Nguyen H, Van Gerven N, Slättegård R, Hernalsteens J‐P, et al. Intervening with urinary tract infections using anti‐adhesives based on the crystal structure of the FimH–Oligomannose‐3 Complex. PLoS One 2008;3:e2040. 10.1371/journal.pone.0002040 18446213 PMC2323111

[98] Cusumano CK, Pinkner JS, Han Z, Greene SE, Ford BA, Crowley JR, et al. Treatment and prevention of urinary tract infection with orally active FimH inhibitors. Sci Transl Med. 2011;3:109ra115. 10.1126/scitranslmed.3003021 PMC3694776 22089451

[99] Hung CS, Bouckaert J, Hung D, Pinkner J, Widberg C, DeFusco A, et al. Structural basis of tropism of *Escherichia coli* to the bladder during urinary tract infection. Mol Microbiol. 2002;44:903–15. 10.1046/j.1365-2958.2002.02915.x 12010488

[100] Ofek I, Mirelman D, Sharon N. Adherence of *Escherichia coli* to human mucosal cells mediated by mannose receptors. Nature 1977;265:623–5. 10.1038/265623a0 323718

[101] Kranjcec B, Papes D, Altarac S. D‐mannose powder for prophylaxis of recurrent urinary tract infections in women: a randomized clinical trial. World J Urol. 2014;32:79–84. 10.1007/s00345-013-1091-6 23633128

[102] Robinson J. D‐mannose to prevent recurrent urinary tract infections. ISRCTN. Accessed 27/02/2020, 10.1186/ISRCTN13283516

[103] Spaulding CN, Klein RD, Ruer S, Kau AL, Schreiber HL, Cusumano ZT, et al. Selective depletion of uropathogenic *E. coli* from the gut by a FimH antagonist. Nature 2017;546:528–32. 10.1038/nature22972 28614296 PMC5654549

[104] Sharon N. Carbohydrates as future anti‐adhesion drugs for infectious diseases. Biochim Biophys Acta. 2006;1760:527–37. 10.1016/j.bbagen.2005.12.008 16564136

[105] Kearney CJ, Vervoort SJ, Ramsbottom KM, Todorovski I, Lelliott EJ, Zethoven M, et al. SUGAR‐seq enables simultaneous detection of glycans, epitopes, and the transcriptome in single cells. Sci Adv. 2021;7:eabe3610. 10.1126/sciadv.abe3610 33608275 PMC7895430

